# Monthly Summary

**Published:** 1895-07

**Authors:** 


### Monthly Summary.

THE ADMINISTRATION OF CHLOROFORM.—Richard Gill, in the last volume of Saint Bartholomew's Hospital has an article entitled "Notes on Chloroform Anæsthesia," which, as it is based on large experience, will be read with interest. In conclusion Mr. Gill asks the question: "What is the best sign to be guided by, when chloroform is administered?" He comments thereon as follows:

"This is a question which does not admit of a general answer, because different rules have to be obeyed during the induction of anæsthesia, during long and short anæsthesia, and also when alterations are made in the patient's position. This much, however, may be stated concerning the induction of chloroform-anæsthesia: the pulse may be ignored, but the respiration must be assiduously watched. How do we know that the patient is unconscious? By the alteration which the breathing undergoes; it is no longer controlled by the will; it has become automatic. But the patient being unconscious, how do we measure the amount of chloroform which is safe for the purposes of continued anesthesia? By the size of the pupil. Sometimes the pupil is large and sometimes small, when unconsciousness is induced. The large pupil may mean narcosis; the small pupil is always the sure sign of safe anesthesia. The small, contracted or pin-point pupil is, then, the measure of anesthesia, and is unquestionably the safest sign to trust; for during anesthesia, and especially during prolonged anesthesia, the respiration may become imperceptibly labored, and its new character may escape detection, the more particularly by experienced administrators.

"But while the breathing is becoming impeded, the pupil is slowly dilating and losing its sensitiveness. This change in its character indicates that the extreme limit of anesthesia has been exceeded. The stage of narcosis has been entered upon, and it is the pupil, not the respiration that has given the signal. When the patient's position is altered, as from the supine to the lateral, the breathing becomes obviously changed. To rely entirely on the respiration would, in this new condition, lead to a return of consciousness. Here the pulse is of no use in determining the right amount of chloroform. Is the pulse of no further service? Undoubtedly. In prolonged operations it is the first to give signs of depression, and must be watched with care and attention, so that when it begins to fail, those means may immediately be put into action which are best known to maintain the flagging energies of the patient. It is thus collapse is anticipated, and, if not prevented, in some degree mitigated."—*Journal American Medical Association.*

---

THE INFLUENCE OF PREGNANCY ON CARIES.—Ruben Peterson, M. D., of Grand Rapids, Mich., read a paper with this title before the Grand Rapids Dental Society. *We* cannot do better than copy the doctor's own admirable summary of his observations: 1. It is probably true that dental caries is more liable to occur during pregnancy. 2. Dental caries is a disease characterized by a molecular disintegration of the normal constituents of the teeth. 3. The disease is caused by the action of certain pathogenic micro-organisms which produce lactic acid, which, in turn, decalcifies the enamel and exposes the dentine to the attacks of the bacteria. 4. It is improbable that lime salts are abstracted from the teeth to supply the needs of the growing fœtus. 5. More than enough phosphates are ingested to supply the needs of mother and child, hence the maternal teeth do not suffer from lack of nutrition. 6. During gestation osteophytes are found, showing an excess of lime salts in the system. 7. The true explanation must be looked for in some change in the oral secretions which thereby furnish a more favorable soil for the development of the mioro-organisms. 8. There is evidence to prove that the saliva is more acid during pregnancy. 9. This condition is probably due to the changes in the blood by which its alkilinity is diminished. 10. The analogy between this and the lithemic condition is striking. 11. Vomiting of pregnancy, while it may to some extent aid, cannot be considered a potent factor in the production of caries. 12. Neglect of the teeth during pregnancy cannot be proved to be more prevalent than at other times, and therefore should not be considered among the causes of caries.--*Dental Cosmos.*

---

### THE SANITARY SIN.

**The latest sensational discovery is the unsanitary kiss.**

I met my modest Minnie by the wind-mill in the trees;
We walked among the lillies, those that neither toil nor spin
But a demon danced between us, and he howled above the breeze.
"You may love the little lady, her affections you may win,
But kissing is a sanitary sin."

I married her one morning in the church-yard down the lane,
Far away from the trouble and the turmoil and the din:
Ere the earth had made its journey round the orb of day again
We'd a baby girl, but never did we kiss our little Min,
For kissing is a sanitary sin.

Oh, we love each other dearly, but our lips have never met,
Though her silver threads are coming and my thatch is growing thin;
To the act of osculation we have not decided yet,
For we know, since fell diseases such a practice ushers in,
That kissing is a sanitary sin.

—*British Medical Journal.*

ANTRAL EMPYEMA OF TUBERCULOUS ORIGIN—A case of antral empyema, apparently of tuberculous origin, is recorded by J. Kekwick, in the *British Journal of Dental Science* of May 15. The patient, a woman aged 30, complained of the usual symptoms indicative of antral empyema. The left upper second bicuspid was extracted and a free opening into the antrum made through the socket. For twelve months local treatment, combined with change of air and the administration of tonics, was carried out, but to no avail, the patient's condition remaining practically unchanged. The pus being of a curdy character and the history of the patient led to a suspicion of tubercle, etc., and on the discharge being examined microscopically, the tubercle bacillus was found in large quantities. Constitutional treatment for tubercle, combined with the insufflation into the antrum every day of powdered iodoform, led to rapid improvement in the patient's condition, the discharge becoming less and the hectic condition which the patient had commenced to acquire being lost. The following reasons are given in support of the diagnosis: (1) The chronic course of the case, with no local causes such as loose sequestra; (2) the tuberculous character of the pus; (3) the amenability of the disease to iodoform; (4) the history of the patient (uncle died from phthisis; sisters suffering from phthisis, no signs of tubercle in the patient herself, but a queried history of tuberculous cervical glands—cicatrices); and (5) the bacilla in the pus which was washed directly out of the antrum through the nose.

---

DEAD TEETH.—"We often hear about 'dead teeth,' but I am very much opposed to the use cf the term 'dead tooth' unless the tooth is really dead. The term is unscientific in the majority of cases, and sounds badly to the patient. If a person thinks he has a dead tooth in his mouth he is apt to look on it with abhorrence and doubt its usefulness. If we call such teeth 'pulpless,' no such unfavorable impression is given, and we are far more accurate in our statement"—Dr. Potter in *Dominion Dental Journal.*

